# Real-Time Sensing of Output Polymer Flow Temperature and Volumetric Flowrate in Fused Filament Fabrication Process

**DOI:** 10.3390/ma15020618

**Published:** 2022-01-14

**Authors:** Rakshith Badarinath, Vittaldas Prabhu

**Affiliations:** Department of Industrial and Manufacturing Engineering, The Pennsylvania State University, University Park, PA 16802, USA; rakshith.badarinath@psu.edu

**Keywords:** fused filament fabrication, additive manufacturing, real-time sensing, melt temperature estimation, polymer flowrate, vision-based measurement, robotics, image processing, response surface design

## Abstract

In this paper we addressed key challenges in engineering an instrumentation system for sensing and signal processing for real-time estimation of two main process variables in the Fused-Filament-Fabrication process: (i) temperature of the polymer melt exiting the nozzle using a thermocouple; and (ii) polymer flowrate using extrusion width measurements in real-time, in-situ, using a microscope camera. We used a design of experiments approach to develop response surface models for two materials that enable accurate estimation of the polymer exit temperature as a function of polymer flowrate and liquefier temperature with a fit of $R^{2}=99.96\%\;\mathrm{and}\;99.39\%$. The live video stream of the deposition process was used to compute the flowrate based on a road geometry model. Specifically, a robust extrusion width recognizer $\left(\mathrm{REXR}\right)$ algorithm was developed to identify edges of the deposited road and for real-time computation of extrusion width, which was found to be robust to filament colors and materials. The extrusion width measurement was found to be within 0.08 mm of caliper measurements with an $R^{2}$ value of 99.91% and was found to closely track the requested flowrate from the slicer. This opens new avenues for advancing the engineering science for process monitoring and control of FFF.

## 1. Introduction

Fused filament fabrication (FFF) continues to be among the most widespread additive manufacturing (AM) processes for making polymeric functional prototypes and, in several cases, end-use parts. At present, FFF is most widely used because of its economic appeal and capability to process a variety of thermoplastics, and in scales large enough to fabricate car bodies, boat hulls, and large tooling [1,2]. In an FFF process, a thermoplastic feedstock, usually available as spooled filament, is fed into the hot-end portion of an extrusion system, where it is heated, melted, and forced through a nozzle. The resulting molten polymer, called a road, is deposited to complete a cross-section of the part, and the stacking of consecutive cross-sections results in the final three-dimensional part. Some of the current challenges in industrializing AM are variability in product quality and the need for rapid qualification of parts. In-situ process monitoring via process sensing and process control has recently gained a lot of attention from researchers and is the most important venue for realizing the challenge of industrialization and the development of robust AM processes. Continuous sensing of key process parameters not only helps to better understand the physics of the FFF process but can also help detect process drifts and track product quality in-situ. When combined with process control, online adjustment of process parameters can potentially aid in process corrections leading to consistent product quality and reduced material wastage due to failure avoidance. Furthermore, with recent interest in sustainable manufacturing involving efforts to develop green composite feedstocks for FFF using recycled materials and agricultural waste [3], wherein properties of the feedstock and thus the resulting part could vary within a part and between batches, the need for process monitoring becomes ever more vital.

**Figure 1:**
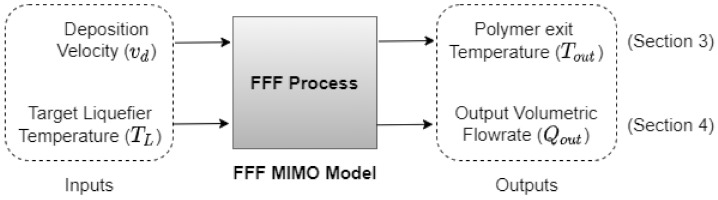
FFF multi-input multi-output (MIMO) model to represent process dynamics.

The dynamics of the FFF process can be viewed as a two-input, two-output system as shown in Figure 1. In the FFF process, the melt temperature of the polymer exiting the nozzle $\left(T_{out}\right)$ is a function of heat input into the liquefier block, the rate at which filament is fed into the extruder $\left(v_{E}\right)$ for a given filament material properties including thermal conductivity, density, and specific heat capacity. It is important to note that in FFF systems, the deposition velocity $\left(v_{d}\right)$, i.e., the velocity of the motion system, is directly coupled with $v_{E}$ and usually dictates $v_{E}$ for a required road geometry. The liquefier temperature $\left(T_{L}\right)$ is the temperature of the liquefier block inside which the polymer melts and is typically well-controlled in FFF printers through a PID control. The liquefier block houses the heater element, nozzle, and a temperature sensor that is typically mounted axially along the length of the nozzle. The liquefier temperature ($T_{L}$) directly affects $T_{out}$ and it is intuitive that the higher the $T_{L}$, the higher the $T_{out}$ will be. Additionally, for a fixed $T_{L}$, studies have shown that $T_{out}$ decreases with increasing $v_{E}$ [4]. This is due to the fact that as $v_{E}$ increases, the residence time and thus time for the polymer to melt shortens, reducing $T_{out}$. In extreme cases when $v_{E}$ exceeds the hot-end melt capacity, the backpressure and thus the feeding force increase exponentially within the nozzle, leading to skipped steps or nozzle jams [5]. Similarly, the output volumetric flowrate $\left(Q_{out}\right)$ from the nozzle, also referred to as “polymer flowrate” in this paper, is also dependent on $T_{L}$ and $v_{E}$. For a fixed $T_{L}$, increasing $v_{E}$ increases $Q_{out}$ according to the road geometry model [6]. At higher $v_{E}$ where under-extrusion is present, increasing $T_{L}$ for the same $v_{E}$ reduces the viscosity of the molten polymer, back pressure in the nozzle, and leads to increased polymer flowrate. 

$T_{out}$ is an important process variable to monitor in real-time as it directly influences the inter-layer bond strength and thus the macro-mechanical properties of the part being printed [7,8,9,10]. There have been few notable efforts to measure the polymer melt temperature and pressure inside the nozzle to better understand the physics of the FFF process. Coogan et al. [11] developed an in-line rheometer consisting of load cell and thermocouple to measure viscosity, pressure, and melt temperature of the polymer flow inside the nozzle. Several corrections were applied to the raw data such as correcting for entrance effects to obtain accurate viscosity measurements. In a similar effort, Anderegg et al. [12] developed a new nozzle design consisting of a pressure transducer and a thermocouple to measure the temperature and pressure of the polymer melt. A 6.5 °C drop in melt temperature was observed at the higher flowrates even with a calibrated PID temperature loop. Most of the temperature sensors used today in FFF printers are either thermistors or thermocouples and, due to the nature of these temperature sensors, the resulting temperature measurements are only accurate in the installation location. Most extruder designs feature temperature sensors that are usually not in direct contact with the nozzle or are mounted co-axial or far away from the nozzle. In these cases, the temperatures measured by temperature sensors are not representative of actual molten polymer temperature within the nozzle and that exiting the nozzle. Some studies have shown that these temperature differences can be up to 20 °C [13]. Furthermore, the reviewed literature on thermal modeling of melt flow within the liquefier [14,15,16] fails to adequately account for radial and axial variations in temperature field within liquefier, leading to discrepancies in experimentally measured values of temperatures, pressures, and feeding forces [4]. This is also a result of limited available measurements of the temperature profile in the liquefier. 

Another method commonly used for temperature measurement is non-contact infrared (IR) imaging where the accuracy of the measurement depends on the emissivity. Based on existing literature, an IR camera is used for measuring spatial and temporal temperature profiles in the printed part for process monitoring [17,18], to understand strength evolution and influencing process parameters [10,19,20], and to validate analytical models for part strength prediction [9]. However, when measuring nozzle temperatures using an IR camera, it is important to note that the emissivity of the metal nozzle changes when heated and the surface becomes contaminated quickly during the printing process. Therefore, IR cameras are rarely used for measuring the temperature of the nozzle in commercial FFF 3D printers.

It is not feasible to obtain contact-based temperature measurements of the molten polymer exiting the nozzle and hence non-contact measurements based on IR imaging can be used. Unfortunately, the IR temperature measurements are limited by line of sight, which makes it impractical for production environments. Furthermore, IR sensors and associated instrumentations are far more expensive and bulkier than thermocouples. Therefore, in this paper, we used a design of experiments approach to develop a response surface model that enables accurate estimation of $T_{out}$ as a function of inputs: $T_{L}$ and $Q=\left(\frac{\pi d_{f}^{2}}{4}\times v_{E}\right)$, where $d_{f}$ is the diameter of the filament, and is valid for the entire operating space of the FFF process, using an IR camera-based $T_{out}$ measurements and contact-based nozzle thermocouple measurements. 

For the in-situ measurement of the polymer flowrate $\left(Q_{out}\right)$, we used a vision-based approach to first measure the extrusion width $\left(w\right)$ during deposition, and then calculate $Q_{out}$ from w using road geometry model [6]. There are several studies on using vision-based techniques for process monitoring and defect detection for the FFF process. Fang et al. [21] implemented an online process-monitoring system to detect the processing defects during FFF-based ceramic components fabrication using process signature extracted from layer-wise optical imaging. Cheng and Jafari [22] used image intensity transformations to extract defect signatures from 2D layer-wise images and classified defects into two types: randomly occurring defects and anomalies. The defects were correlated to flowrate control to minimize such defects in the printed part. In related work, Liu et al. [23] demonstrate an online image-based closed-loop quality control for identification and correction of over-fill and under-fill defects during FFF using 2D layer-wise images. A textural analysis-based image classification algorithm was used for defect detection and a PID feedback controller was used for adjusting printer parameters. Additionally, statistical process control methods such as quality charts have been used to evaluate the quality of layer contours using 2D layer-wise images obtained using machine vision [24,25]. Furthermore, there are studies in the existing literature that attempt to (i) reconstruct layer contours in 3D using single or multiple camera setups to identify defects [26,27,28], (ii) use laser scanners for 2D defect detection [29], and (iii) use deep learning methods (e.g., CNN) for 2D defect detection and classification [30].

However, none of the above literature attempt to “measure” road geometry in-situ. Additionally, all the studies deal with layer-wise images or 3D images and not imaging at the road geometry level. It is important to note that the dimensions at road level dictate layer-wise and overall part-wise quality. To the best of our knowledge, this is the first work that attempts to measure extrusion width and resulting volumetric flowrate in-situ during deposition. This work aimed for in-situ real-time estimation of $T_{out}$ and measurement of w and $Q_{out}$ process variables in the FFF process during deposition. The experimental testbed, including details on sensor instrumentation and signal flows, is described in Section 2. The IR imaging approach to estimate $T_{out}$ is discussed in Section 3. In Section 4, the vision-based approach to measure extrusion width is presented in detail along with validation of the measurements. Conclusions and future work are discussed in Section 5. 

## 2. Experimental Testbed

**Figure 2:**
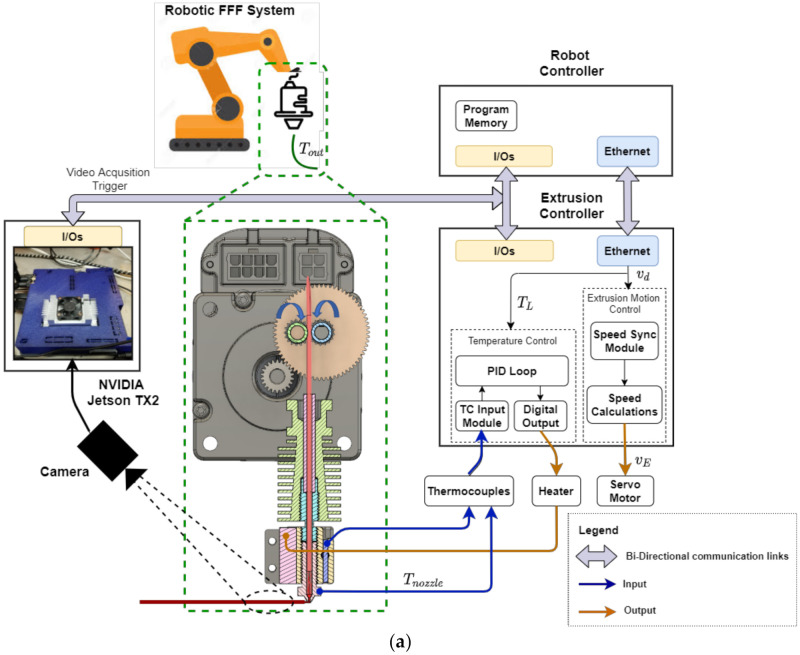

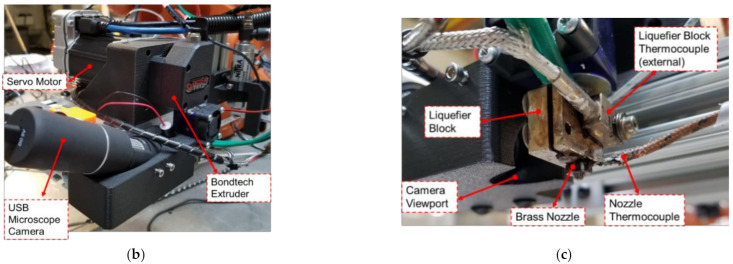
(**a**) Overview of architecture and signal flow for the robotic FFF testbed; (**b**) Updated robotic FFF extrusion system with Servo motor, Bondtech extruder, and USB microscope camera; (**c**) Closer view at the hot-end portion of the extruder showing the installed nozzle thermocouple.

In our previous work on integration and evaluation of robotic FFF system [6], we engineered a fully functional research testbed in which integration and real-time synchronization of robot motion and extrusion controller was achieved by (1) communicating space-variant process parameters in real-time using TCP/IP sockets, and (2) analog and digital I/O interfacing to ensure that the extrusion velocity and deposition velocity matched closely by building upon an analytical process model. To further gain a fundamental understanding of the FFF process, we instrumented it with sensors. A new thermocouple was installed at the nozzle hex region that can provide accurate temperature measurements of polymer melt within the nozzle. A current sensor was installed to measure current consumption by the resistive heating element in the hot-end. A schematic of the instrumented experimental testbed and associated signal flow is illustrated in Figure 2a. Figure 2b,c show the experimental system used in this paper consisting of a Bondtech BMG extruder [31] that features dual gripping gears and a closed-loop servo motor with torque feedback capability [32]. These changes allow for improved reliability of the developed robotic FFF system and help monitor filament feed force through torque feedback. Additionally, the extrusion system was also instrumented with a microscope camera [33] to measure road geometry during printing and is discussed in detail in Section 4. The video acquisition using the camera for in-situ extrusion width measurement was triggered by a digital I/O signal between PLC and the Nvidia Jetson TX2 board. The timing of when to trigger and stop video acquisition was embedded into the robot program at the time of program generation by considering the $\left(x,y\right)$ coordinates of the path to be printed.

## 3. Output Polymer Flow Temperature Estimation

### 3.1. Experimental Setup

**Figure 3:**
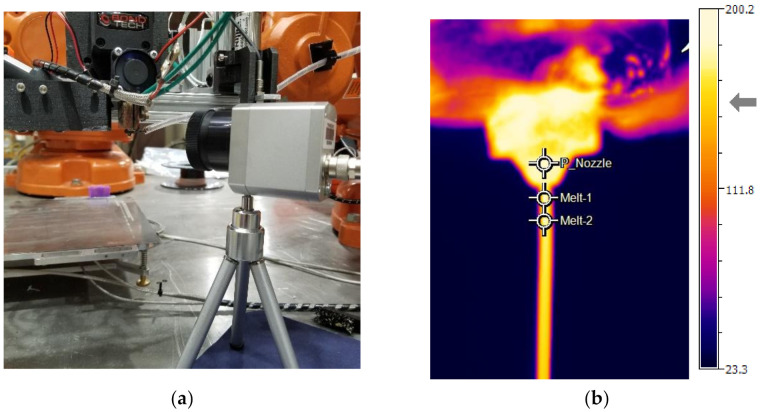

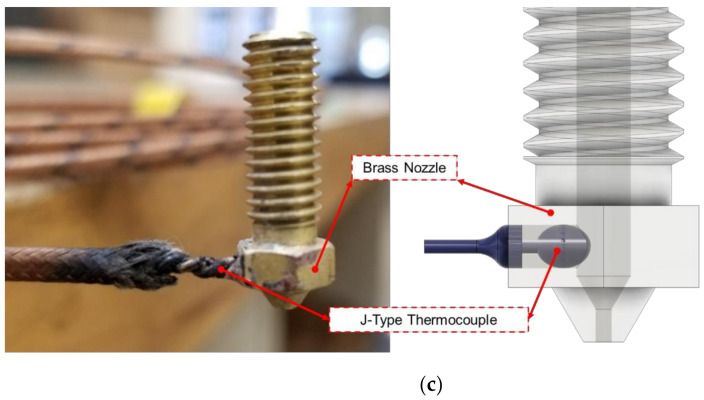
(**a**) IR camera setup for measurement of the polymer exit temperature (**b**); IR image showing locations of crosshairs; (**c**) Modified brass nozzle with thermocouple.

The experimental setup for estimating the temperature of the molten polymer immediately exiting the nozzle is shown in Figure 3a. The polymer exit temperature was measured by IR imaging using an Optris Pi 400 IR camera [34] and Optris PIX Connect process monitoring software (rel. 3.2.3023.0) [35]. The IR camera allows for a measurement speed of 80 Hz and has an optical resolution of 382 × 288 pixels. The resulting IR image containing locations of temperature measurement through crosshairs is shown in Figure 3b. The crosshair ‘Melt-1” was set to measure the polymer immediately exiting the nozzle and is the temperature of interest. To calibrate and validate IR measurements, the 0.6 mm brass nozzle was modified to accommodate a thermocouple as shown in Figure 3c. A ϕ1.60-mm hole was drilled through the hex portion of the nozzle and the tip of the J-type thermocouple wire [36] was inserted into the nozzle and fastened in place using silver solder. Care was taken to not insert the tip of the thermocouple into the internal channel of the nozzle, which could obstruct the polymer flow. With this setup, the tip of the thermocouple measures molten polymer temperature inside the nozzle.

The first step in acquiring reliable temperature readings from the IR camera is to calibrate the emissivity value using a known reference. Given the short distance (<3 mm) between the location of the nozzle thermocouple and nozzle opening, the emissivity value was adjusted such that $T_{\mathrm{out}}$ measured at crosshair “Melt-1” (placed immediately below the nozzle opening) using the IR camera was equal to the temperature measured by the nozzle thermocouple ($T_{\mathrm{nz}}$). This method of calibration resulted in an emissivity value of 0.82. It is infeasible to measure $T_{\mathrm{out}}$ by placing a thermocouple immediately below the nozzle opening, as it leads to a build-up of molten polymer on the thermocouple and flow obstruction. A secondary emissivity calibration method based on data in the existing literature was used. The emissivity value of 0.92 was used for molten polymer (PLA and PETG) [37,38]. The results of DOE using both emissivity calibration methods are provided in Section 3.3.

### 3.2. Experimental Design

Designed experiments were used to predict the temperature of the molten polymer immediately exiting the nozzle as a function of input volumetric Flowrate ($Q_{\mathrm{in}}$) and liquefier temperature ($T_{L}$). $Q_{\mathrm{in}}$ is primarily driven by $v_{E}$, i.e., $Q_{\mathrm{in}}=\frac{\pi d_{f}^{2}}{4}\times v_{E}$ where $d_{f}$ is the filament diameter. A central composite response surface design with alpha = 1, i.e., face-centered design was chosen.

Table 1 summarizes the list of factors and their levels. For a face-centered design, axial points are at the center of each face of the factorial space and the design required three levels of each factor. The “Mid” level was calculated as the mid-point between high and low levels. Additionally, the high level for Q was selected such that it was close to the maximum rated value for the extruder and there were no inconsistencies in polymer flow at $T_{L_{\mathrm{Low}}}$. This selection of factor levels ensures that the entire feasible FFF process space is evaluated and the resulting output temperature ($T_{\mathrm{out}}$) estimation covers the entire operating region. There was a total of 28 runs, including two replicates per factor-level combination, and all runs were randomized. The experiment was carried out for two different materials, PLA and PETG, to understand material-dependent temperature influence on $T_{\mathrm{out}}$.

**Figure 4:**
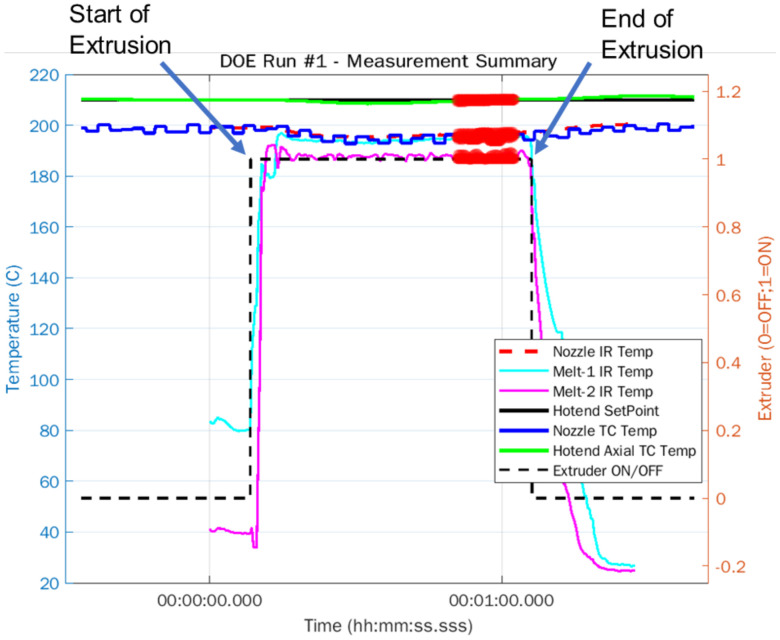
Temperature time diagram constructed using IR and thermocouple temperature data showing various temperatures during step input test. Red regions are the time-series data selected in the stable regime for analysis.

With the IR camera mounted at a fixed distance from the extruder, as shown in Figure 3a, molten polymer was extruded in free air for all runs in the experimental design for a period of 60 s, which is an adequate time to measure steady-state temperature dynamics. The extended period of extrusion mitigated the influence of non-steady state extrusion, e.g., overheating of residual polymer between runs and pressure build-up in the nozzle. The temperature at the “Melt-1” crosshair was recorded vs. time as a temperature-time diagram log in Optris PIX Connect software. Simultaneously, the thermocouple temperatures were also logged in the PLC. Finally, to obtain the value of the response variable $T_{\mathrm{out}}$, a region of steady state temperatures was chosen and averaged, as illustrated in Figure 4. 

### 3.3. Results and Discussion

**Figure 5:**
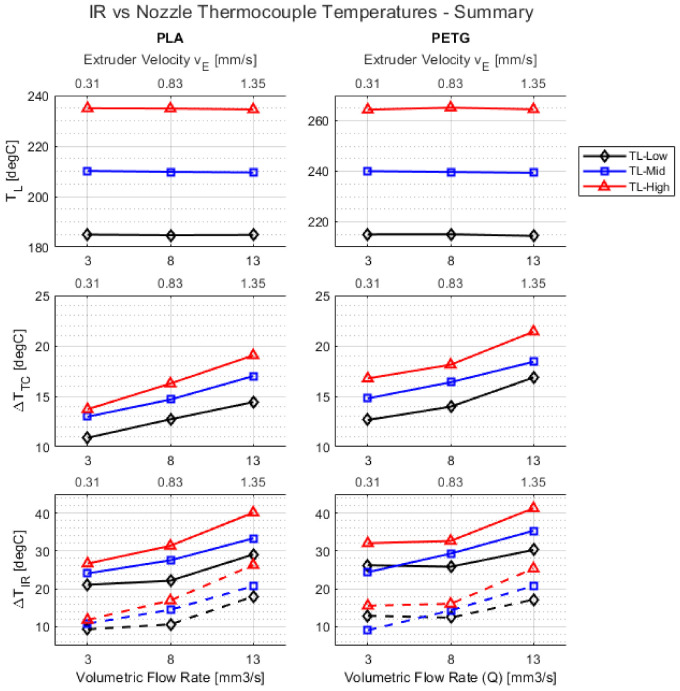
Summary of IR and nozzle thermocouple measurements averaged for all combinations of DOE runs. $\Delta T_{TC}=T_{L}-T_{\mathrm{nz}}$ and $\Delta T_{IR}=T_{L}-T_{IR}$. The dotted lines in the $\Delta T_{IR}$ plots represent $T_{\mathrm{out}}=T_{IR}=T_{\mathrm{nz}}$ measured with emissivity e=0.82 calibrated based on the nozzle thermocouple while solid lines represent $T_{\mathrm{out}}=T_{IR}$ measured with emissivity e=0.92 and calibrated based on existing literature. $T_{L_{\mathrm{Low}}}$, $T_{L_{\mathrm{mid}}}$, and $T_{L_{\mathrm{high}}}$ are based on Table 1.

Figure 5 summarizes the temperature measurements obtained using the IR camera and nozzle thermocouple for all combinations of DOE runs. Key observations that can be made are as follows:

(1) $T_{L}$ is the temperature measured by the axial thermocouple in the liquefier block and it remained constant irrespective of $v_{E}$. This demonstrates that the PID temperature loop was tuned correctly and resulted in excellent tracking of $T_{L}$ with commanded temperatures.
(2) $\Delta T_{TC}=T_{L}-T_{\mathrm{nz}}$ is the temperature difference between liquefier temperature measured by axial thermocouple and nozzle temperature measured by installed nozzle thermocouple. Similarly, $\Delta T_{IR}=T_{L}-T_{IR}$ is the temperature difference between the liquefier temperature measured by axial thermocouple and temperature of the polymer exiting the nozzle measured using the IR camera. There was a general trend of ΔT increasing with increasing $v_{E}$. This is due to the shortened residence time of polymer within the nozzle as $v_{E}$ increases. Additionally, ΔT increased with increasing $T_{L}$. This showed an increasing temperature gradient in the liquefier block because of larger convective heat losses with increasing $T_{L}$ and the entire heat input was not necessarily absorbed by the nozzle. This could be potentially minimized through some insulation of the liquefier block.
(3) There was an average of a 15 °C difference between $T_{L}$ and $T_{\mathrm{nz}}$, i.e., the polymer melt temperature within the nozzle was 15 °C colder than commanded liquefier temperature for all combinations of $T_{L}$ and $v_{E}$. This temperature difference should be accounted for in the commanded liquefier temperature to achieve desired melt viscosity.
(4) $\left(T_{\mathrm{out}}=T_{\mathrm{nz}})>(T_{\mathrm{out}}=T_{\mathrm{IR}}\right)$, i.e., nozzle thermocouple measurements tend to slightly overestimate $T_{\mathrm{out}}$ compared with IR camera temperatures. This is potentially due to the nozzle thermocouple being mechanically fastened to the walls of the conductive brass nozzle.
(5) There was an average of a 25 °C difference between $T_{L}$ and $T_{\mathrm{out}}$ measured using the IR camera.
(6) Even though PETG has a higher melting temperature compared with PLA, the average temperature difference $\Delta T_{IR}$ was roughly the same for both materials.

Finally, for analysis of the DOE, $\Delta T_{IR}$ with e=0.92 values are used.

**Figure 6:**
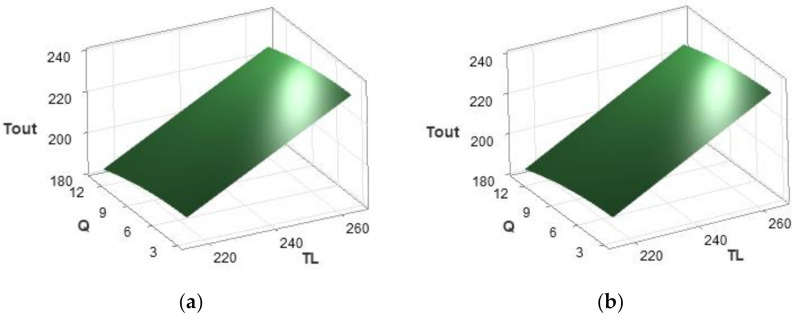
Results of response surface analysis for predicting $T_{out}$ for (**a**) PLA (**b**) PETG.

Equations (1) and (2) describe the resulting response surfaces that can be used for predicting $T_{\mathrm{out}}$ as a function of Q and $T_{L}$ for PLA and PETG and which had excellent fits of $R^{2}$ = 99.96% and $R^{2}$ = 99.39% respectively. Figure 6a,b shows the relationship between the response variable $T_{\mathrm{out}}$ and two predictor variables (Q and $T_{L}$) as a 3D surface plot for PLA and PETG respectively.

(1) $$T_{out\;\left(PLA\right)}=-5.98+0.9187\times T_{L}+2.361\times Q-0.06197\times Q^{2}-0.01159\times T_{L}\times Q$$

(2) $$T_{out\;\left(PETG\right)}=10.00+0.8414\times T_{L}-0.433\times Q-0.0801\times Q^{2}$$

## 4. Vision-Based Polymer Flowrate Estimation

An in-situ vision-based approach was used to measure the extrusion width during printing. For this purpose, the extruder tool assembly was instrumented with a USB microscope camera mounted at an angle and pointed towards the nozzle as shown in Figure 2. (a) Overview of architecture and signal flow for the robotic FFF testbed; (b) (a). The camera can record videos at 20 frames per second (FPS) and each frame has a native resolution of 1280 × 720 pixels.

### 4.1. Camera Calibration 

**Figure 7:**
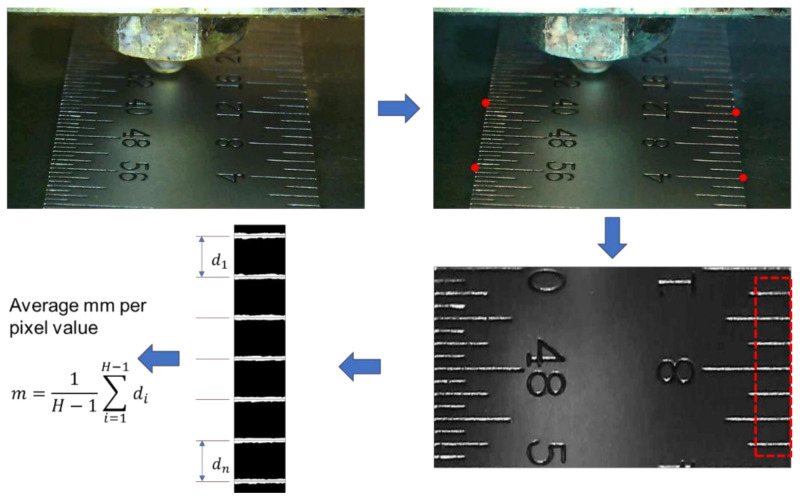
Sequence of steps in mm-per-pixel calibration procedure starting from top left, original image, 4-point selection, perspective corrected image, binary image of scale graduations (H = total number of hatch marks in the image).

To measure extrusion width using the camera, a calibration procedure was performed to determine the “mm per pixel” value which is used to convert image pixels into a physical distance measurement. The camera calibration procedure is illustrated in Figure 7 and involves measuring the number of pixels between two features in an image whose physical distance is well defined and known (e.g., a ruler). A metal ruler with fine hatch marks was used as a calibration device. Since the camera is mounted at an angle and the resulting image acquired by the camera is skewed, the first step is to correct for image perspective using the *warp transform* technique [39]. The original skewed image was corrected for warp using *warp transform*, which is a perspective transform method that requires four non-collinear points on the input-skewed image and known distances between points to compute a 3 × 3 transformation matrix. Open-source computer vision library *OpenCV* [40] was used for all the image processing steps in this section. Next, the perspective corrected output image was converted into a grayscale image, filtered using a 5 × 5 gaussian kernel to denoise, and then converted to a binary image using *Otsu’s thresholding technique* [41,42]. The image was then re-sized to contain the only region of interest (ROI), i.e., scale hatch marks. Finally, Table A1 provides the pseudocode of the algorithm to identify and compute the mean distance between hatch marks to obtain mm per pixel value. Using this calibration method, the average mm-per-pixel value was computed to be 0.004992.

### 4.2. In-Situ Vision-Based Extrusion Width Measurement

**Figure 8:**
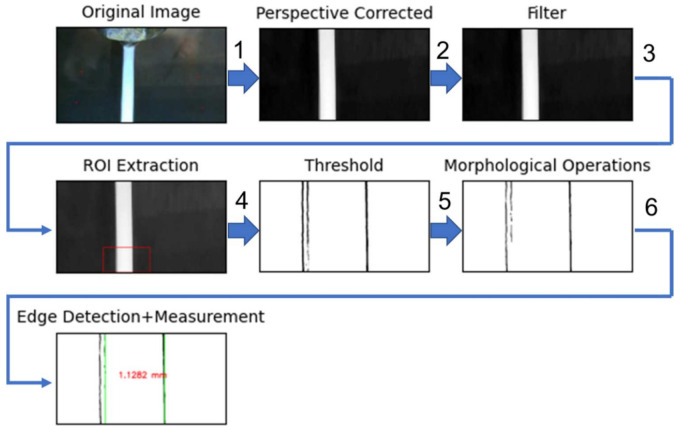
Sequence of image processing steps used and corresponding algorithms-(1) four-point perspective correction, (2) Bilateral Filtering, (3) Image re-sizing, (4) Adaptive Gaussian Thresholding, (5) Multi-pass morphological closing, (6) REXR algorithm.

**Figure 9:**
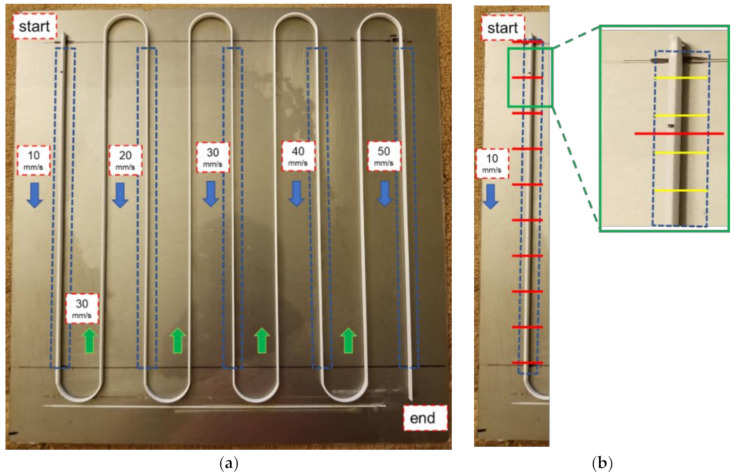
(**a**) Test specimen for in-situ vision-based extrusion width measurement consisting of deposition paths of varying speeds highlighted in a blue dashed rectangle (200 mm). (**b**) Caliper measurement scheme at red intervals (20 mm). Yellow lines are locations of repeatability measurements in the vicinity of a single red interval.

To measure the extrusion width using the camera during printing, the continuous video feed from the camera was processed through a sequence of image processing operations as illustrated in Figure 8. A test specimen consisting of continuous paths with regions of varying deposition speeds was printed to evaluate the performance of the vision-based extrusion width measurement approach and is shown in Figure 9. The single wall test specimen was printed using White PLA material at a layer height of 0.20 mm and was 20 layers tall (4 mm). The specimen consisted of five equal-length (200 mm) regions that were printed at varying deposition speeds starting from 10 mm/s up to 50 mm/s incrementing 10 mm/s. Due to the location of the camera, as shown in Figure 2a, the output polymer flow and thus the resulting road formation could only be seen through the camera when the extruder tool moved from front to back on the build plate. The road of the previous layer could be viewed when the extruder tool moved in the opposite direction. The five deposition paths with varying speeds in the test specimen were designed to be printed from the front of the build plate to the back for this purpose.

#### 4.2.1. Sequence of Image Processing Operations to Measure Extrusion Width

Each frame in the video sequence was processed through a series of image processing operations to identify road edges and measure the extrusion width in real-time. The initial sequence of operations is similar to that used in the camera calibration procedure. In the last stage, an algorithm was developed for robust road edge detection and extrusion width measurement and is explained in Section 4.2.2. All the image processing operations were run on an Nvidia Jetson TX2 embedded hardware board as shown in Figure 2a. First, the image frame in the video sequence was corrected for perspective using the *four-point warp transform* method that used the same 3 × 3 transformation matrix obtained during camera calibration, resulting in a normal top-down view of the image of the deposited road. Next, the perspective corrected image was converted to grayscale and filtered using *bilateral filtering* [43], which is effective in noise removal while keeping edges sharp by taking into account intensity difference between the pixels for determining the filter weights. An ROI was chosen from the filtered image to speed up image processing and was then converted into a binary image using the *adaptive gaussian thresholding* method [41]. The resulting binary image contained not so well-defined edges of the road, usually consisting of noisy pixels and holes in the foreground object. To obtain a clearer definition of the road edges, morphological operations such as *dilation* and *erosion* [44] were performed in that sequence, which is also known as the *closing* operation to remove small holes inside the foreground extrusion width and small black points on the road. Rectangular kernels of size 8×2 and 5×2 were used for dilation and erosion operations respectively. Finally, the resulting binary image was processed using an algorithm to accurately identify edges of the current road and measurement of extrusion width, which is described next.

#### 4.2.2. Robust Extrusion Width Recognizer (REXR) Algorithm

Starting with the binary image $B\in {\mathbb{R}}^{m\times n}$ of the deposited road obtained from the sequence of image processing operations up to morphological closing operation discussed in the previous section, the REXR algorithm scanned the image first across all the columns in a given row (along n direction) before moving onto the next row of pixels (along the m direction). Any transition from a white pixel to a black one could be a starting pixel of a potential road edge and vice versa. Let $N\in {\mathbb{R}}^{m\times 1}$ represent the column vector of the number of edges found across all n columns and for each row m. e represents the maximum number of edges (white to black transitions) found across all the rows of the image, i.e., $e=\max\left(N\right)$. Let $Es\in {\mathbb{R}}^{m\times e}$ denote the matrix containing pixel locations where there is a white to black transition, i.e., $B_{i,j}=0$, and $Ee\in {\mathbb{R}}^{m\times e}$ denote the matrix containing pixel locations where there is a black to white transition, i.e., $B_{i,j}=255$. 

Typically, a well-defined image of a road should have two edges, but in real conditions, there are cases where the image could have more than two edges. For example, if the extrusion width of the currently deposited layer is narrower than that of the previously deposited layer, the edges of the previous layer will be visible in the image and the total number of edges in the image may exceed two. Additionally, there could be noisy black pixels that could result in “pseudo-edges”. The REXR algorithm aims to identify the correct set of edges of the road being deposited even in cases of poor edge definition (i.e., missing edge pixels or discontinuity in edges). The algorithm computes starting and ending pixel coordinates of the left and right edges of the road, i.e., $e_{left}\left(x_{1},y_{1}\right),\;\;e_{left}\left(x_{1},y_{2}\right),\;\;e_{right}\left(x_{2},y_{1}\right),$ and $e_{right}\left(x_{2},y_{2}\right)$, using Es,  Ee, and e. The x coordinates of $e_{left}$ and $e_{right}$ represent the average pixel location of the road edges. In cases where e=2, the x coordinates of $e_{left}$ and $e_{right}$ are given by Equations (3) and (4) respectively.

(3) $$e_{left}\left(x_{1}\right)\;=\frac{1}{2}\overset{m}{\underset{i=1}{\sum }}k1_{i}+k2_{i}\;\forall \;k1\in Es_{m,1}\left[e\right];\;k2\in Ee_{m,1}\left[e\right]\;where\;e=2$$

(4) $$e_{right}\left(x_{2}\right)\;=\frac{1}{2}\overset{m}{\underset{i=1}{\sum }}k1_{i}+k2_{i}\;\forall \;k1\in Es_{m,2}\left[e\right];\;k2\in Ee_{m,2}\left[e\right]\;where\;e=2\;$$

Finally, the extrusion width (w) is given by Equation (5)

(5) $$w=e_{right}\left(x_{2}\right)-e_{left}\left(x_{1}\right)$$

In cases where e>2 due to reasons described above, “true” left and right road edges were computed using Es and Ee matrices using Equations. For case e=3, the start pixel locations of “true” left” and right edges were calculated using Equations (6) and (7). The end pixel locations of “true” left and right edges ($\hat{Ee_{l}}$ and $\hat{Ee_{r}}$) were also computed using Equations (6) and (7) by replacing Es with Ee.

(6) $$\hat{Es_{l}}=\;\left[k\right]\;\forall \;k\in \left\{Es_{m,1}\right\}\;where\;e=3\;and\;Es_{i,j}<\frac{n}{2}$$

(7) $$\hat{Es_{r}}=\;\left[k\right]\;\forall \;k\in \left\{Es_{m,1}\right\}\;where\;e=3\;and\;\frac{n}{2}<Es_{i,j}<\frac{3n}{4}$$

The x coordinates of $e_{left}$ and $e_{right}$ were calculated based on pixel locations in vectors $\hat{Es_{l}}$ and $\hat{Es_{r}}$ according to Equations (8)–(15).

(8) $$e_{left}\left(x_{1}\right)=\left\{\begin{array}{c} \;A\;if\;\hat{Es_{l}}\neq \emptyset \;AND\;\hat{Es_{r}}=\emptyset \;AND\;length\left(\hat{Es_{l}}\right)>e_{th}\;\\ B\;if\;\hat{Es_{l}}=\emptyset \;AND\;\hat{Es_{r}}\neq \emptyset \;AND\;length\left(\hat{Es_{r}}\right)>e_{th\;}AND\;e=3\\ C\;if\;\hat{Es_{l}}=\emptyset \;AND\;\hat{Es_{r}}\neq \emptyset \;AND\;length\left(\hat{Es_{r}}\right)>e_{th\;}AND\;e\geq 2 \end{array}\right.$$

(9) $$A=\frac{1}{2}\sum (\hat{Es_{l}}+\hat{Ee_{l}})$$

(10) $$B=\frac{1}{2}\overset{p}{\underset{i=1}{\sum }}k1_{i}+k2_{i}\;\forall \;k1\in Es_{m,1}\left[e\right];\;k2\in Ee_{m,1}\left[e\right]\;where\;e=3$$

(11) $$C=\frac{1}{2}\overset{p}{\underset{i=1}{\sum }}k1_{i}+k2_{i}\;\forall \;k1\in Es_{m,1}\left[e\right];\;k2\in Ee_{m,1}\left[e\right]\;where\;e=2$$

(12) $$e_{right}\left(x_{2}\right)=\left\{\begin{array}{c} \;D\;if\;\hat{Es_{l}}\neq \emptyset \;AND\;\hat{Es_{r}}=\emptyset \;AND\;length\left(\hat{Es_{l}}\right)>e_{th}\;AND\;e=3\;\\ E\;if\;\hat{Es_{l}}\neq \emptyset \;AND\;\hat{Es_{r}}=\emptyset \;AND\;length\left(\hat{Es_{l}}\right)>e_{th}\;AND\;e\geq 2\\ F\;if\;\hat{Es_{l}}=\emptyset \;AND\;\hat{Es_{r}}\neq \emptyset \;AND\;length\left(\hat{Es_{r}}\right)>e_{th\;} \end{array}\right.$$

(13) $$D=\frac{1}{2}\overset{p}{\underset{i=1}{\sum }}k1_{i}+k2_{i}\;\forall \;k1\in Es_{m,3}\left[e\right];\;k2\in Ee_{m,3}\left[e\right]\;where\;e=3$$

(14) $$E=\frac{1}{2}\overset{p}{\underset{i=1}{\sum }}k1_{i}+k2_{i}\;\forall \;k1\in Es_{m,2}\left[e\right];\;k2\in Ee_{m,2}\left[e\right]\;where\;e=2$$

(15) $$F=\frac{1}{2}\sum (\hat{Es_{r}}+\hat{Ee_{r}})$$

Finally, for the case e=4, the same set of Equations could be used to compute “true” left and right edges with an additional scenario when both $\hat{Es_{l}},\;\hat{Es_{r}}\neq \emptyset$. In that case, vectors $\hat{Ee_{l}}$ and $\hat{Ee_{r}}$ were also computed using Equations (6) and (7) using elements where e=4. Cases where e>4 typically consist of regions of noisy non-significant edges whose pixel length is less than the threshold $e_{th}$, which can be eliminated to identify true edges. In all cases of e, the extrusion width (w) was calculated according to Equation (5). 

### 4.3. Validation of Vision-Based Extrusion Width Measurements

To validate the extrusion width measurements obtained using vision, the extrusion width of the printed test specimen was also measured using calipers along the length of the deposition path. Measurements were taken at an equal interval of 20 mm over the total length (200 mm) of the deposition region as shown in Figure 9a. Furthermore, five measurements were taken in the vicinity of a single 20 mm interval for repeatability as illustrated using yellow lines in Figure 9b. This resulted in a total of 50 caliper measurements per one 200 mm long deposition path. There was a total of five such deposition paths in the test specimen, one for each deposition speed, i.e., 10 mm/s to 50 mm/s. A link to the video illustrating the working of the vision-based extrusion width measurement approach in real time and associated code is provided in the Supplementary Materials section of this paper. 

**Figure 10:**
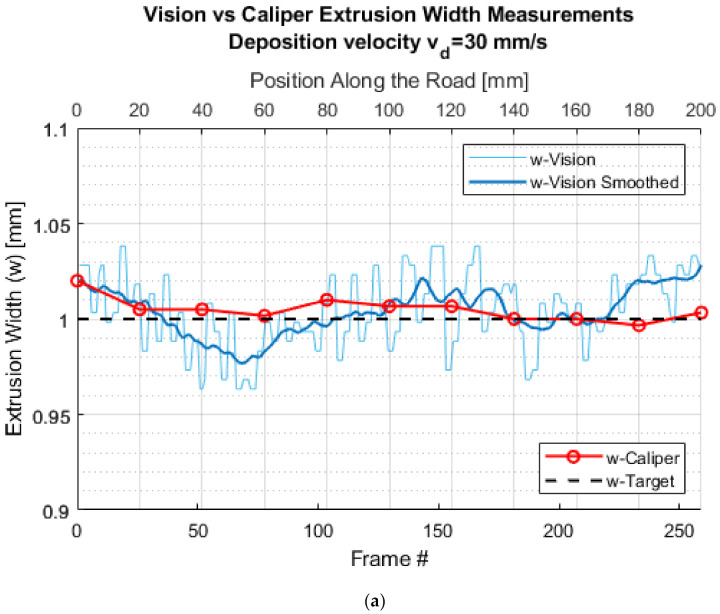

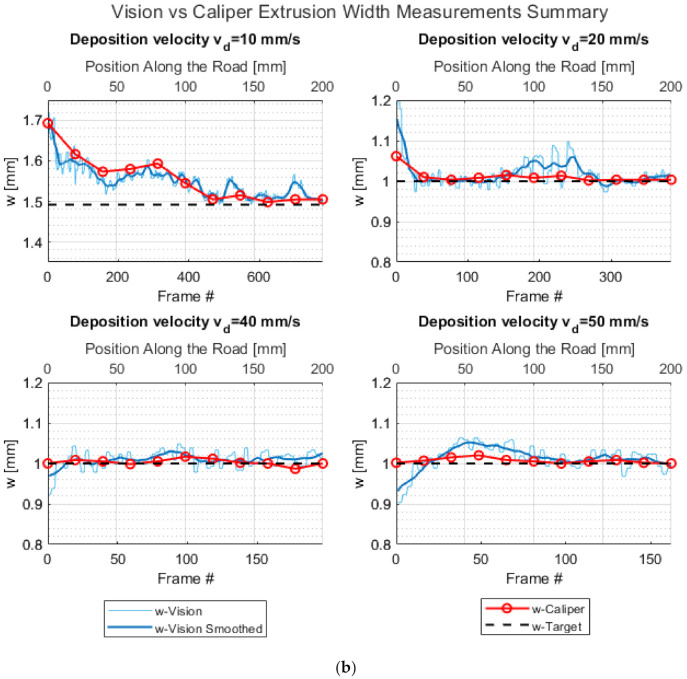
Validation of the vision-based extrusion width measurements with caliper-based measurements for various deposition speeds (**a**) 30 mm/s, (**b**) Summary of measurements for 10, 20, 40 and 50 mm/s. Note: w-Target is the designed wall thickness.

Figure 10 show the results of the vision-based extrusion width measurement in comparison to caliper measurements and target or slicer specified extrusion width for five different deposition speeds. The *X*-axis in each figure represents the frame number in the video sequence. All the extrusion width measurements were taken using vision for the last (i.e., 20th) layer. To denoise and smoothen the vision-based extrusion width measurements computed for each frame of the video sequence, a moving averaging filter with a window size of 20 samples was used. As evident from Figure 10, the vision-based extrusion width measurements were in very good agreement with caliper measurements with an average error of less than 0.08 mm.

**Figure A1:**
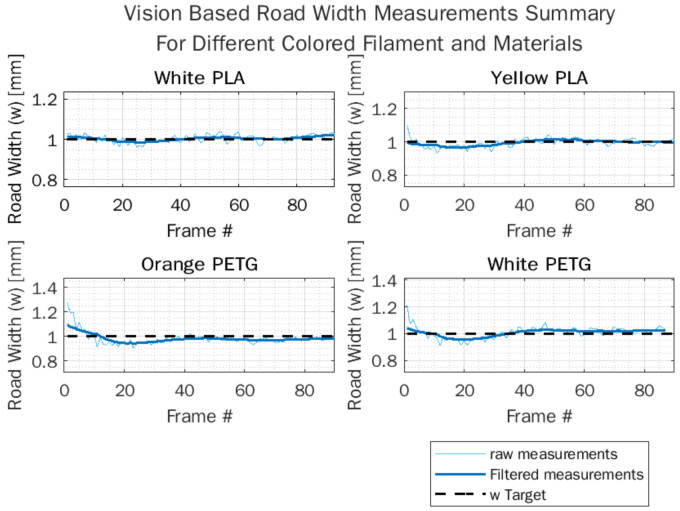
Summary of vision-based extrusion width measurements for different colored filaments and materials.

**Figure A2:**
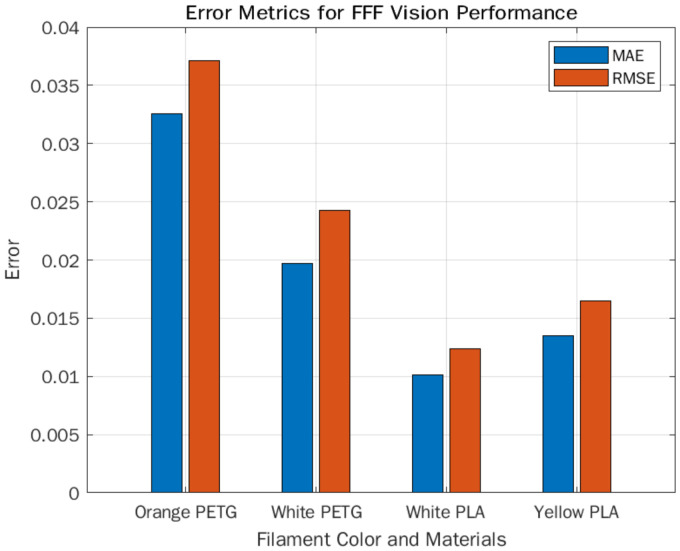
Mean Absolute Error (MAE) and Root Mean Square Error (RMSE) metrics for performance evaluation of FFF vision for different colored filaments and materials.

Additionally, the robustness of the vision-based extrusion width measurement approach was evaluated for various filament colors and polymer materials and the results are provided in Appendix A.2. As evident from Figure A1, the tested filament colors and materials did not affect the measurement accuracy of the extrusion width. Figure A2 provides error metrics (MAE and RMSE) for the performance of the vision-based extrusion width measurement for different color filaments and materials. With maximum MAE and RMSE values of 0.0325 mm and 0.0371 mm respectively, the vision-based measurement approach can be confidently used for real-time process monitoring.

### 4.4. Polymer Flowrate Estimation

Using vision-based extrusion width $\left(w\right)$ measurements, it is possible to calculate the output volumetric flowrate $\left(Q_{out}\right)$ using the road geometry model. In our previous work on robotic FFF System [6], we presented and experimentally validated the road geometry model that is based on the flow guidelines provided by the open-source slicing software “slic3r” for modeling the resulting road geometry [45]. In the FFF process, a thermoplastic filament of diameter $\left(d_{f}\right)$ fed into the hot-end at a rate $\left(v_{E}\right)$ defined by the deposition head velocity $\left(v_{d}\right)$ and pushed out of a nozzle with a circular orifice with a diameter $\left(d_{n}\right)$ results in a rectangular road with semi-circular ends [46]. The road takes this geometry when molten plastic out of the nozzle is deposited onto the bed, or a previous layer separated by a small distance equal to layer height $\left(h\right)$.

The cross-sectional area of the deposited road is given by,

(16) $$A_{road}=\left(w\times h\right)-\left(\left(1-\frac{\pi }{4}\right)\times h^{2}\right)=\left(\left(w-h\right)\times h\right)+\left(\pi \times \frac{h^{2}}{4}\right)$$

The output volumetric flowrate $\left(Q_{out}\right)$ can be calculated using road cross-sectional area $\left(A_{road}\right)$ and deposition velocity $\left(v_{d}\right)$ as follows,

(17) $$Q_{out}=A_{road}\times v_{d}$$

The average layer height $\left(\hat{h}\right)$ is measured using a custom robot-based dial indicator probing method wherein the overall height of the test specimen is measured at the same caliper measurement points and is divided by the number of layers. Typically, the actual layer height will be the same as the commanded layer height $\left(h\right)$, i.e., $\hat{h}≅h$ because layer height is largely dictated by the positioning accuracy of the *Z*-axis motion system. Moreover, as shown in our previous work [6], bed leveling and compensation algorithms can be effectively used to account for any deviations in print bed surface to achieve the requested road geometry. 

**Figure 11:**
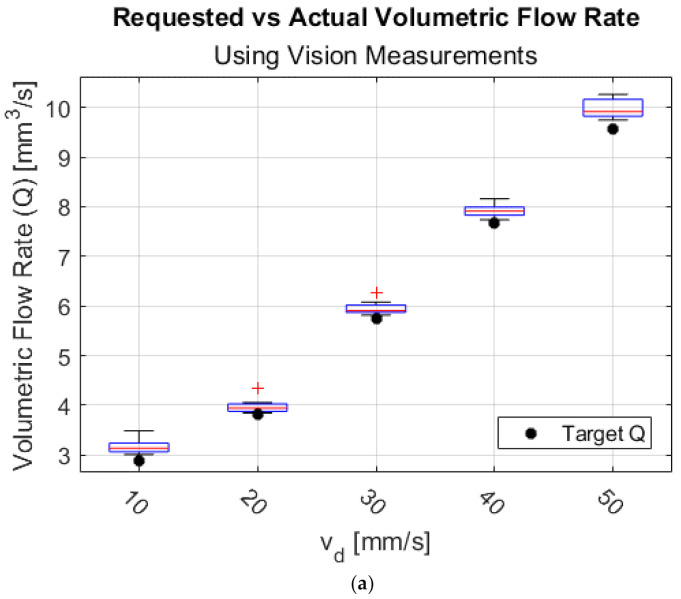

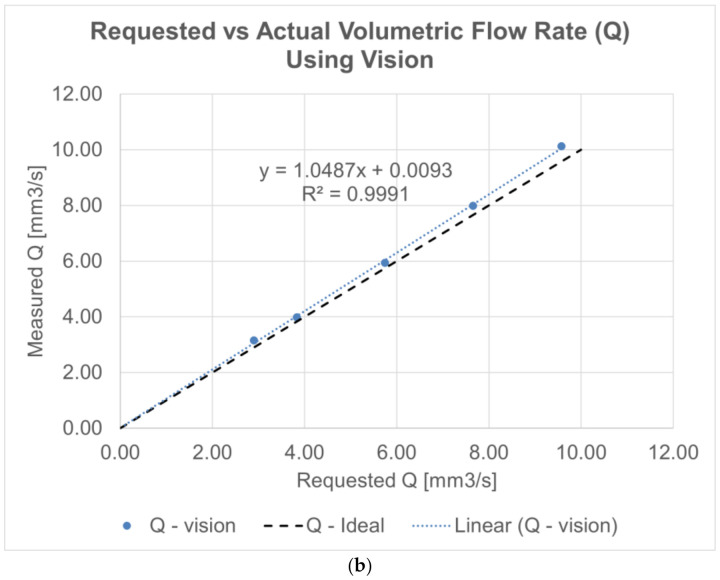
Output volumetric flowrate calculated using vision-based extrusion width measurements and part height measurements. (**a**) Box plot showing for $Q_{out}$ for different deposition velocities; (**b**) $Q_{out}$ calculated using vision (blue line) vs. requested from the slicer. The dotted black line shows an ideal case where measured $Q_{out}$ = requested $Q_{out}$.

Figure 11a shows a box plot of $Q_{out}$ calculated using w for different $v_{d}$. Figure 11b plots the actual volumetric flowrate $\left(Q_{Actual}\right)$ vs. requested volumetric flowrate $\left(Q_{requested}\right)$ for the entire tested operating region of the FFF process. The dotted black line represents the ideal case of volumetric flowrate conservation assumption, i.e., $\left(Q_{in}=Q_{out}\right)$, resulting in a 45° line. $Q_{Actual}=Q_{out}$ (blue line) was computed according to Equation (17) using average measured values for w and h. With an $R^{2}$ value of 99.91%, the $Q_{out}$ calculated vision-based extrusion width measurement is in agreement with the requested volumetric flowrate $\left(Q_{requested}\right)$ and demonstrates that $Q_{out}$ can be reliably measured using the vision approach using the proposed REXR algorithm. 

### 4.5. Frame Processing Time Considerations

**Figure 12:**
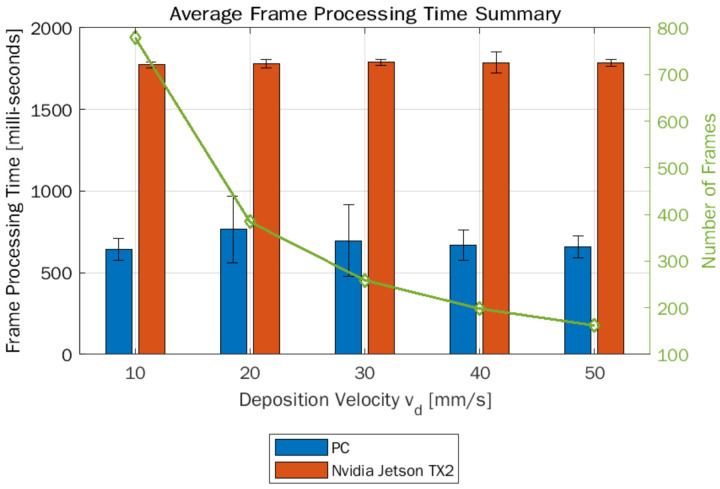
Summary of frame processing time (FPT) for in-situ extrusion width measurement.

An important consideration in deploying vision-based measurement techniques, in addition to measurement accuracy, is the achievable measurement sampling rate, i.e., how quickly measurements can be made. In general, all video cameras have a metric to describe the acquisition rate called “frames per second (FPS)”, but the measurement sampling rate will be limited by the computing time required for image processing. The camera used in this study had a rated video acquisition rate of 20 FPS; however, based on experimental data, the actual achievable video acquisition rate was found to be 13–14 FPS. To evaluate the average time required to process a single frame and to determine the measurement sampling rate achievable, the complete sequence of image processing steps described in Section 4.2.1 and Section 4.2.2 were timed during execution on the Nvidia Jetson TX2 board and PC with an Intel Core i5-7300U CPU + 8 GB RAM. Figure 12 shows the average frame processing times (FPT) on PC and Nvidia board along with the number of frames available for different deposition velocities in the test specimen. As the deposition velocity increased, the length of the video acquisition and the number of frames decreased for the same deposition path length. The vision-based extrusion width measurement approach took about 0.687 s on PC and 1.78 s on the Nvidia board to process a single frame, resulting in a sampling rate of about 1.45 and 0.56 samples per second, respectively. Finally, as evident from Figure 12, the FPT remained the same irrespective of deposition velocity. 

## 5. Conclusions and Future Work

In this paper, we engineered an instrumentation system consisting of sensors and signal processing for real-time estimation of two main process variables in the FFF process: (i) temperature of the polymer melt immediately exiting the nozzle $\left(T_{out}\right)$ and (ii) output volumetric flowrate $\left(Q_{out}\right)$, also referred to as “Polymer flowrate”, using extrusion width $\left(w\right)$ measurements. We designed experiments to develop a response surface model for estimation of $T_{out}$ as a function of process inputs: deposition velocity $\left(v_{d}\right)$ and liquefier temperature $\left(T_{L}\right)$ and for two materials: PLA and PETG, which is valid over the entire operating region of the FFF process. A regular 0.6 mm brass nozzle was modified to include a thermocouple in the hex region where polymer melting occurs. The temperature measured using the nozzle thermocouple was used for emissivity calibration of IR-based temperature measurements of polymer exiting the nozzle. The resulting emissivity value differed from ones available in existing literature, indicating the nozzle thermocouple measures a mixture of nozzle wall and polymer temperatures. On average, the temperature of the polymer immediately exiting the nozzle was found to be 15 to 20 °C colder than the temperature reported from the stock thermocouple in the hot-end. The resulting response surface models can be used for estimating $T_{out}$ as a function of input filament volumetric flowrate (Q) and $T_{L}$ for PLA and PETG materials and had excellent fits of $R^{2}$ = 99.96% and 99.39% respectively. For estimating $Q_{out}$, we used a vision-based approach utilizing a continuous video sequence from a USB microscope camera to measure w during deposition. A sequence of image processing operations available in the OpenCV library was applied to each frame of the video sequence to correct for camera orientation, denoising, ROI extraction, and binarization. An algorithm called “robust extrusion width recognizer” $\left(\mathrm{REXR}\right)$ was developed to accurately identify road edges and measure extrusion width from binary images. Vision-based extrusion width measurements obtained during printing of a single-walled test specimen with regions of varying deposition velocity showed excellent agreement with caliper measurements with a maximum error of 0.08 mm and were robust to tested filament colors and materials. Finally, $Q_{out}$ computed based on the road geometry model using measured w and h values had a fit of $R^{2}=99.91\%$ and closely matched the requested volumetric flow rate from the slicer. 

One of the future research opportunities that arises from this work is to use the real-time estimations of process variables for feedback process control to improve part quality. While the measurement rate of vision-based extrusion width is acceptable for process monitoring, it needs to be improved for real-time control. Additionally, due to the location of the camera, extrusion width in the blind-spot regions during deposition could not be measured at this time. This could be addressed by having multiple cameras or a camera positioning mechanism around the nozzle. Our future and ongoing work will address these and other advances.

## Figures and Tables

**Table 1:** Experimental design setup for output melt temperature prediction.

Factors	Symbol	Levels	
		Low	High
Target Liquefier Temperature (°C)	$T_{L}$	PLA: 185	235
		PETG: 215	265
Volumetric Flowrate (${\mathrm{mm}}^{3}/s$)	Q	3	13
**Materials**			PLA and PETG
**Design Type**			Central Composite (α=1) Face centered
**Blocks**			2
**Replicates**			2

## Data Availability

The data presented in this study are available on request from the corresponding author.

## Supplementary Materials

Developed code for vision-based extrusion width measurement is available on GitHub: https://github.com/rakshithb/FFF-Vision, accessed on 13 December 2021; Video of vision-based extrusion width measurement approach on YouTube: https://youtu.be/pUR17u_Hyds, accessed on 13 December 2021.

## List of Abbreviations

CNN	Convolutional Neural Networks
CV	Computer Vision
FFF	Fused Filament Fabrication
FPS	Frames Per Second
FPT	Frame Processing Time
I/O	Input/Output
MIMO	Multi-Input Multi-Output
PETG	Polyethylene terephthalate glycol
PID	Proportional Integral Derivative
PLA	Polylactic Acid
PLC	Programmable Logic Controller
ROI	Region of Interest
TCP/IP	Transmission Control Protocol/Internet Protocol
USB	Universal Serial Bus

## Appendix A

### Appendix A.1. Pseudocode for Camera Calibration

**Table A1:** Pseudocode to compute average mm/pixel value for camera calibration.

**Pseudocode to Compute Average mm/pixel Value**
Input: Binary image of scale graduations; size: m×n Output: average mm/pixel value 1. Scan the image first across rows and then across columns. A hatch start has a black to white pixel transition while a hatch end has the opposite transition. Obtain ma-trices containing locations of hatch mark start and end: $h_{s}\;\left\{size:\;\left(H\times n\right)\right\}$ and $h_{e}\;\left\{size:\;\left(H\times n\right)\right\}$. Compute center of hatch marks using start and end matrices $h_{c}\;\left\{size:\;\left(1\times H\right)\right\}$ where H is the total number of hatch marks found in the image. FOR i in range (number of columns in the binary image) ∣ RESET index k and hatch marks counter H ∣ FOR j in range (number of rows in the binary image) ∣ ∣ IF pixel value at location $\left[i,j\right]\;\geq$ 250 ∣ ∣ ∣ IF found_hatch_flag IS FALSE ∣ ∣ ∣ ∣ SET found_hatch_flag to TRUE ∣ ∣ ∣ ∣ record location of hatch start: $h_{s}\left[i\right]\left[k\right]\;=j$ ∣ ∣ ∣ ∣ INCREMENT index k ∣ ∣ ∣ ∣ INCREMENT hatch marks counter H ∣ ∣ ∣ END IF ∣ ∣ ELSE (i.e., check if a black pixel is found) ∣ ∣ ∣ IF found_hatch_flag IS TRUE ∣ ∣ ∣ ∣ record location of hatch end: $h_{e}\left[i\right]\left[k-1\right]\;=j-1$ ∣ ∣ ∣ ∣ SET found_hatch_flag to FALSE ∣ ∣ ∣ END IF ∣ ∣ END IF ∣ END FOR ∣ Compute center of hatch marks $h_{c}\left[i\right]\;=\frac{1}{2}\sum_{l=1}^{H}\left(h_{s}\left[i,l\right]+h_{e}\left[i,l\right]\right)$ END FOR 2. Let $h_{c}=\;\left[h_{c}{}_{1},h_{c}{}_{2},h_{c}{}_{3},\ldots \;,h_{c}{}_{H}\right]$ then the distance between the center of hatch marks is $d=\;\left[d_{1},d_{2},\ldots ,\;d_{H-1}\right]\;=\;\left[h_{c}{}_{i+1}-h_{c}{}_{i}\right]\;\forall \;i=1,2,\ldots ,H\;$ 3. The average mm-per-pixel value is the average of all elements in 1-D row vector d.
